# Effects of multidimensional exercise management on self-efficacy, blood glucose control, and delivery outcomes in pregnant women with gestational diabetes mellitus

**DOI:** 10.3389/fphys.2024.1407569

**Published:** 2024-08-16

**Authors:** Ying He, Xin Liu, Xiali Yang

**Affiliations:** Nursing College, Ningxia Medical University, Yinchuan, China

**Keywords:** multidimensional quantitative exercise management, gestational diabetes mellitus, self‐efficacy, blood glucose, delivery outcomes

## Abstract

**Objective:**

This study aimed to evaluate the effects of multidimensional quantitative exercise management on self-efficacy, blood glucose control, and delivery outcomes in pregnant women with gestational diabetes mellitus (GDM).

**Methods:**

A randomized controlled trial was conducted with 150 pregnant women diagnosed with gestational diabetes mellitus (GDM). Participants were randomly assigned to either the experimental group (Exp), which received a multidimensional quantitative exercise management intervention, or the control group (Con), which received standard GDM management. Results were compared between the groups included self-efficacy scores, blood glucose levels, and delivery outcomes.

**Results:**

Exp group of pregnant women exhibited drastically superior self-efficacy scores as well as more stable blood glucose levels during pregnancy relative to Con group (*P* < 0.05). Moreover, visual analogue scale (VAS) of pregnant women and Apgar scores of infants in Exp group were considerably better than those in Con group (*P* < 0.05). In contrast to Con group, pregnant women in Exp group had considerably better labor outcomes along neonatal complication rates (*P* < 0.05).

**Conclusion:**

Multidimensional quantitative exercise management had a positive impact on pregnant women with GDM. This intervention method can improve self-efficacy levels along better blood glucose control, and enhance delivery outcomes. These findings suggested that multidimensional quantitative exercise management has potential clinical value in the management of GDM, providing an effective management strategy to improve the health conditions of both pregnant women and infants.

## 1 Introduction

Gestational diabetes mellitus (GDM) is a metabolic condition marked by increased levels of glucose in the blood during pregnancy. It is usually identified after 20 weeks of gestation and affects around 5%–10% of pregnant women globally (Alejandro et al., 2020; Sweeting et al., 2022). The condition arises due to hormonal changes that lead to insulin resistance and insufficient functioning of the pancreatic beta-cells (Juan et al., 2020). Although gestational diabetes mellitus (GDM) typically recovers after childbirth, it does elevate the likelihood of getting diabetes in the future (Craig et al., 2020). Globally, there is a growing prevalence of gestational diabetes mellitus (GDM), with more than 1.6 million new cases reported each year. This increase is attributed to various reasons including obesity, older mother age, unhealthy food, and lack of physical activity (Carrasco-Wong et al., 2020; Vince et al., 2020; Tsakiridis et al., 2021). Gestational diabetes mellitus (GDM) presents considerable hazards to the health of both the mother and the newborn. These dangers include gestational hypertension, preeclampsia, cesarean delivery, macrosomia (excessive birth weight), neonatal hypoglycemia, and respiratory distress syndrome (Karimova et al., 2020; Wu et al., 2020). Research suggests that pregnant women with gestational diabetes mellitus (GDM) are at a higher risk of experiencing complications such as gestational hypertension and placental dysfunction, as compared to pregnancies without diabetes (Chu and Godfrey, 2021). Additionally, there is a greater likelihood of macrosomia (abnormally large babies) occurring in GDM pregnancies, with a prevalence of 24% compared to 8% in non-diabetic pregnancies (Lende and Rijhsinghani, 2020; Wu et al., 2021; Jasińska-Stroschein and Waszyk-Nowaczyk, 2023).

This study aimed to evaluate the impact of multidimensional quantitative exercise management on self-efficacy, blood glucose control, and delivery outcomes in women with GDM. The study compared the effects of a comprehensive exercise management approach, utilizing modern technologies for continuous monitoring and personalized feedback, against traditional GDM management methods.

Efficient management of gestational diabetes mellitus (GDM) is crucial in order to decrease the health risks that are linked to it for both mothers and infants. The conventional care of gestational diabetes mellitus (GDM) mainly consists of regulating the diet, monitoring blood glucose levels, and administering insulin therapy. However, these approaches frequently encounter difficulties such as poor compliance and inconsistent blood glucose regulation (Hewage et al., 2020; Huang et al., 2021). Multidimensional quantitative exercise management is a promising option by integrating customized exercise regimens with real-time monitoring and customized feedback (Feng et al., 2020a; Allman et al., 2022). This strategy seeks to increase motivation and self-confidence, leading to improvements in exercise habits, blood glucose control, cardiovascular health, and delivery outcomes (Valentin and Bernstein, 2020; Guo et al., 2022; Smart et al., 2022; Jasińska-Stroschein and Waszyk-Nowaczyk, 2023). Gaining a comprehensive understanding of self-efficacy, which refers to confidence in individuals in their capability to successfully do particular activities, is of utmost importance in this particular situation. The Pregnancy activity Self-Efficacy Scale (P-ESES) is a reliable instrument utilized to evaluate the self-assurance of pregnant women in their capacity to engage in physical activity (Gielnik et al., 2020; HJNo, 2020; Graham, 2022; Wu et al., 2022). The findings of this study can be used to shape the development of specific interventions aimed at increasing exercise engagement and contentment among pregnant women. This can ultimately lead to a novel and efficient approach for managing gestational diabetes mellitus (GDM) and provide valuable recommendations for healthcare professionals.

## 2 Data and methodologies

### 2.1 General information

This experimental study included 150 GDM pregnant women who were enrolled in prenatal care and delivery at one hospital in Ningxia Hui Autonomous Region from January 2022 to July 2022. The patients were randomly assigned into control (Con) group and experimental (Exp) group, with 75 patients in each group. Con group received standard GDM management, while Exp group received multidimensional quantitative exercise intervention management. Among the 75 patients in Con group, the age ranged from 25 to 32 years, with a mean age of (28 ± 1.4) years. Among the 75 patients in the study group, the age ranged from 24 to 34 years, with a mean age of (29 ± 1.9) years. No statistically marked differences existed in general characteristics between the two groups (*P* > 0.05). The study had been approved by the This experimental study included 150 GDM pregnant women who were enrolled in prenatal care and delivery at Ningxia Medical University from January 2022 to July 2022. Medical Ethics Committee, and informed consent forms had been signed by the patients and their families.

According to Eq. 3, the required sample size for the two groups can be calculated, with *α* = 0.05, *β* = 0.1, and the *U*_*α*_ and *U*_*β*_ can be consulted through referring to corresponding tables (Alejandro et al., 2020; Sweeting et al., 2022).

### 2.2 Inclusion and exclusion criteria

#### 2.2.1 Inclusion criteria

The inclusion criteria for this study were as follows: (1) Age between 20 and 35 years, primigravida with a singleton pregnancy; (2) Gestational age ≥ 24 weeks; (3) Confirmed diagnosis of GDM according to the *Diagnosis and Treatment Guidelines for Gestational Diabetes Mellitus* (2014); (4) Sufficient cognitive ability, no mental abnormalities, and the ability to independently complete the questionnaires; (5) Absence of other metabolic disorders, normal liver and kidney function, no history of major surgeries, and the ability to engage in physical activity independently; (6) Voluntary participation in the study and signing of informed consent forms.

#### 2.2.2 Exclusion criteria

The exclusion criteria for this study were as follows: (1) Use of other hypoglycemic medications such as insulin; (2) Family history of diabetes, hypertension, or other hereditary diseases; (3) Preexisting diabetes prior to pregnancy.

### 2.3 Content and methods of intervention

#### 2.3.1 Con group

Con group received standard care for GDM, which included regular medical check-ups, blood glucose monitoring, dietary control, and recommended daily exercise. During check-ups, patients were educated about exercise precautions. If symptoms such as shortness of breath or dizziness occurred, exercise was immediately stopped, and the patient was advised to rest or consume glucose until recovery. Daily exercise mainly consisted of moderate to low intensity, with a frequency of at least four times per week and a duration of at least 20 min per session, until delivery. The intensity of exercise was self-assessed by the patient and aimed to reach a level of mild sweating and fatigue, while still being able to continue exercising. Detailed records of the exercise regimen were kept for future reference.

#### 2.3.2 Exp group

Exp group received the same standard care for GDM, including regular medical check-ups, blood glucose monitoring, and dietary control. In terms of daily exercise, they received interventions through multidimensional quantitative exercise management. Additional exercise wristbands were provided to monitor parameters such as heart rate during physical activity. The specific measures were as follows:

(1) Establishment of a multidimensional quantitative exercise intervention healthcare team. The team consisted of two obstetricians, two diabetes specialists, four obstetric nurses, two diabetes specialist nurses, and two rehabilitation therapy specialists. All team members underwent unified training on relevant knowledge, including the explanation of the research content, research objectives, specific research methods, review of literature on exercise management for GDM, and international guidelines. They were assessed on their knowledge and had to achieve a passing score to be eligible to participate in the research.
(2) Development of personalized exercise prescriptions. Individualized exercise plans were formulated for each pregnant woman based on their health status and exercise capacity. The assessment of health status relied on medical records prior to pregnancy, pre-pregnancy examinations (including measurements of height, weight, blood pressure, and other basic indicators), and information regarding the woman’s medical history and previous surgeries to evaluate their general health condition. The evaluation of exercise capacity was based on the international physical activity questionnaire (IPAQ) and the 6-minute walk test (6MWT) (Chandrashekaran et al., 2022). The questionnaire, test results, and self-reported exercise habits and preferences of the pregnant women were used to assess their exercise capacity and determine their preferred types of physical activity. Personalized exercise methods were designed with a focus on aerobic exercises of moderate intensity, such as yoga, swimming, and jogging. During exercise, the participants were required to wear exercise wristbands for heart rate monitoring. Daily exercise duration and energy expenditure were recorded, and the data were uploaded to the cloud for statistical backup by the nurses. The recommended exercise frequency was at least once daily, with each session lasting no less than 30 min.
  The determination of exercise intensity is based on the target heart rate (THR) and estimated maximum heart rate (EMHR) (Shookster et al., 2020; Imamura and Kinugawa, 2021). The EMHR refers to the individual’s maximum heart rate calculated using Eq. 1. The THR range is determined based on the individual’s age and health status, indicating the desired heart rate range to be maintained during exercise. Typically, the maximum heart rate is multiplied by a percentage to obtain the THR range, as shown in Eq. 2. Common THR ranges include low-intensity THR range: 50%–70% of the maximum heart rate, moderate-intensity THR range: 70%–85% of the maximum heart rate, high-intensity THR range: 85%–100% of the maximum heart rate.

EMHR=220−AGE (1)

$$THR=EMHR\times THR^{\prime }s\;range$$ (2)

(3) Regular exercise guidance. Regular face-to-face or remote exercise guidance was provided to ensure that pregnant women correctly implemented the exercise plan and addressed any potential questions they may have. In response to the exercise barriers caused by the progression of pregnancy and changes in physical functioning, timely communication with the medical team within the group should be conducted to make corresponding adjustments based on heart rate and other physical indicators. This guidance should continue until the patient gave birth.

### 2.4 Research tools

This study primarily utilized the following tools: the IPAQ, the 6MWT, the P-ESES, the visual analogue scale (VAS), and the Apgar score for newborns.

The IPAQ, developed jointly by the *World Health Organization* and the *International Society for Physical Activity and Health*, is a self-report tool used to assess individual levels of physical activity. It is widely employed in surveys, epidemiological research, and health interventions, demonstrating high reliability and validity (Adanaş Aydın et al., 2020). The IPAQ provides definitions for daily exercise intensity, energy expenditure, and how to calculate levels of physical activity. In this study, the exercise frequency, duration, and other parameters were derived from the IPAQ.

The 6MWT is a commonly used functional test to assess an individual’s cardiorespiratory endurance and walking ability. It was initially proposed and standardized by the American Thoracic Society. In this study, the 6MWT was employed as an auxiliary test to evaluate the activity function of pregnant women. During the test, individuals are required to walk through a standard-length corridor as quickly as possible for a duration of 6 min (Salas-Groves et al., 2023). The distance walked and relevant physiological parameters such as heart rate and respiratory rate are recorded before and after the test.

The P-ESES, developed by Fahrenwald et al., in 2004, is a questionnaire used to assess pregnant women’s self-efficacy beliefs in exercising during pregnancy (Yang et al., 2022). It serves as a self-report tool to understand the level of confidence and competence of pregnant women in engaging in physical activity. The scale has demonstrated high reliability and validity in previous studies. It consists of three dimensions: overcoming barriers to exercise, overcoming emotional barriers, and overcoming support barriers. There are a total of ten items, each rated on a 5-point Likert scale ranging from “strongly disagree” to “strongly agree,” with scores of 1–5, respectively. The total score ranges from 10 to 50, with scores above 40 indicating high self-efficacy, scores of 20 or below indicating low self-efficacy, and scores between 20 and 40 indicating moderate self-efficacy levels.

The VAS is a commonly used measurement tool for subjective assessment of an individual’s sensations, experiences, or pain intensity (Coustan et al., 2021). It typically consists of a straight line with endpoints representing extreme sensations or degrees, ranging from 0 to 10. The individual is required to mark their perceived level of sensation or pain on the line. In this study, VAS was employed as a scoring indicator for assessing labor pain perception.

The Apgar score for newborns is a commonly used tool to evaluate the health status and adaptability of newborns (Gao et al., 2022). It is assessed at 1 min and 5 min after birth, based on indicators such as heart rate, respiration, muscle tone, reflex response, and skin color. Each indicator is scored from 0 to 2, resulting in a total score of 10. In this study, the Apgar score was used to assess the birth conditions of newborns in both groups.

#### 2.4.1 International physical activity questionnaire (IPAQ)

The following questions are about the time you spent engaging in vigorous physical activities during the past 7 days (Table 1). Please only consider activities that lasted for at least 10 minutes. Even if you feel that you are not usually very active, it is important that you try to answer all the questions. You can think about the physical activities you engaged in at home, in parks, at work, or during your leisure time for recreation or exercise.

**TABLE 1:** International physical activity questionnaire (IPAQ).

Question	Description	Response options
1. Vigorous Physical Activities	In the past 7 days, how many days did you engage in vigorous physical activities?	Number of days Or Did not engage in any vigorous activities (Proceed to Question 3)
2. Duration of Vigorous Activities	In the past 7 days, on average, how many hours and minutes per day did you spend on vigorous physical activities?	Average per day: ___ hours ___ minutes Total in the past 7 days: ___ hours ___ minutes
3. Moderate-Intensity Physical Activities	In the past 7 days, how many days did you engage in moderate-intensity physical activities?	Number of days Or Did not engage in any moderate activities (Proceed to Question 5)
4. Duration of Moderate Activities	In the past 7 days, on average, how many hours and minutes per day did you spend on moderate-intensity physical activities?	Average per day: ___ hours ___ minutes Total in the past 7 days: ___ hours ___ minutes
5. Walking	In the past 7 days, how many days did you engage in continuous walking for 10 min or more?	Number of day Or Did not engage in any form of walking (Proceed to Question 7)
6. Duration of Walking	In the past 7 days, on average, how much time did you spend walking per day?	Average per day: ___ hours ___ minutes Total in the past 7 days: ___ hours ___ minutes
7. Sitting	In the past 7 days, excluding weekends, on average, how much time did you spend sitting per day?	Average per day: ___ hours ___ minutes Total on Wednesday: ___ hours ___ minutes

#### 2.4.2 Pregnancy exercise self-efficacy scale (P-ESES)

To facilitate the implementation of exercise during pregnancy for gestational diabetes, we kindly request your cooperation in completing the P-ESES questionnaire (Table 2). Please use the following scale for rating: 1 = Not at all consistent, 2 = Slightly inconsistent, 3 = Moderately consistent, 4 = Quite consistent, 5 = Completely consistent. Please assess your responses based on the given scoring criteria and your personal experience.

**TABLE 2:** Pregnancy exercise self-efficacy scale (P-ESES).

Question	Score
If I try my best, I can overcome any challenges encountered during exercise	1–5
I have confidence in finding suitable ways of exercising that work for me	1–5
I have confidence in meeting my daily exercise goals	1–5
When faced with obstacles during exercise, I have confidence in finding solutions to overcome them	1–5
Even when experiencing fatigue caused by pregnancy, I have confidence in engaging in physical activity	1–5
I have confidence in exercising even when feeling low in mood	1–5
I have confidence in exercising without consulting a doctor	1–5
Even without the support of family and friends, I have confidence in exercising	1–5
After a period of inactivity, I have confidence in resuming exercise	1–5
Even without access to a gym or professional sports facilities, I have confidence in engaging in physical activity	1–5

Scoring: Total scores range from 1 to 5; Scores above 4 indicate high self-efficacy; Scores of 2 or below indicate low self-efficacy; Scores between 2 and 4 indicate moderate self-efficacy levels.

According to reference (Zhao et al., 2020), the rate of meeting the criteria for moderate-intensity exercise in China is 22.6%, i.e., *P*_2_ = 0.226. In this study, a 25% improvement in the intervention group was expected, i.e., *P*_1_ = 0.467. Considering a 20% dropout rate, the calculated sample size was 75.

$$N_{1}=N_{2}=\frac{2P\left(1-P\right){\left(U_{\alpha }+U_{\beta }\right)}^{2}}{{\left(P_{1}-P_{2}\right)}^{2}}$$ (3)

### 2.5 Diagnostic criteria

The diagnostic criteria for GDM used in this study were based on the *Diagnosis and Treatment Guidelines for Gestational Diabetes Mellitus* (2014). Pregnant women who underwent their first examination between 24 and 28 weeks of gestation were subjected to an oral glucose tolerance test (OGTT). They were instructed to consume at least 150 g of carbohydrates for 3 days before the test and fast for 8 h before the examination. A 75 g (300 mL) glucose solution was administered to the patients, which they were required to consume within 5 min. Blood samples were collected from the patients before drinking the solution, as well as 1 h and 2 h after consumption, to measure their blood glucose levels. GDM was ruled out if the levels were less than 5.1 mmol/L, 10.0 mmol/L, and 8.5 mmol/L, respectively. GDM was diagnosed if any one of these measurements exceeded the specified thresholds. Alternatively, fasting blood glucose (FBG) levels were directly measured, and a diagnosis of GDM was made if FBG was greater than or equal to 5.1 mmol/L. If FBG was between 4.4 mmol/L and 5.1 mmol/L, an additional OGTT was performed, and GDM was ruled out if FBG was less than 4.4 mmol/L.

### 2.6 Indicators of observation

(1) The FBG, 2-h postprandial blood glucose, and glycosylated hemoglobin (HbA1c) levels of the two groups of pregnant women were recorded before and 8 weeks after the intervention.
(2) The scores of P-ESES were recorded for both groups of pregnant women before and 8 weeks after the intervention.
(3) The delivery outcomes of both groups of pregnant women were recorded, including the incidence of weight gain, gestational infections, and abnormal amniotic fluid. The incidence of complications in newborns was also recorded.

Specific weight gain ranges were determined as follows: for BMI < 18.5, the expected weight gain was 12.5–18 kg; for 18.5 ≤ BMI < 24.9, the expected weight gain was 11.5–16 kg; for 24.9 ≤ BMI < 29.9, the expected weight gain was 7–11.5 kg; and for BMI ≥ 29.9, the expected weight gain was 5–9 kg (Lewandowska et al., 2020).

(4) The VAS scores for both groups of pregnant women and the Apgar scores for newborns were recorded.

### 2.7 Statistical analysis

Data analysis was performed using SPSS 26.0. Expressed as mean ± standard deviation (*x* ± *s*), continuous variables were analyzed using the *t*-test for between-group comparisons. Presented as frequencies or rates, categorical variables were analyzed using the chi-square test for between-group comparisons. *P* < 0.05 was considered statistically notable, indicating a marked difference.

## 3 Results

### 3.1 Analysis of P-ESES during pregnancy

**FIGURE 1:**
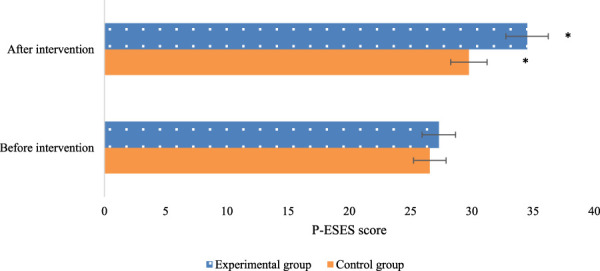
Comparison of P-ESES scores before and after intervention in the two groups of patients (Note: * indicates marked difference).

Comparison of P-ESES scores between the experimental (Exp) and control (Con) groups showed no marked differences before the intervention (*P* > 0.05). Nevertheless, after the intervention, marked differences were observed within each group, with drastically superior scores relative to the pre-intervention scores. This indicates that timely exercise can enhance pregnant women’s self-efficacy and confidence levels toward physical activity. Furthermore, when comparing between the groups, the Exp group demonstrated drastically superior P-ESES scores relative to the Con group (*P* < 0.05). These findings highlight the notable advantage of the multidimensional quantitative exercise management in the Exp group over the conventional GDM management in the Con group. Detailed data are presented in Figure 1.

**FIGURE 2:**
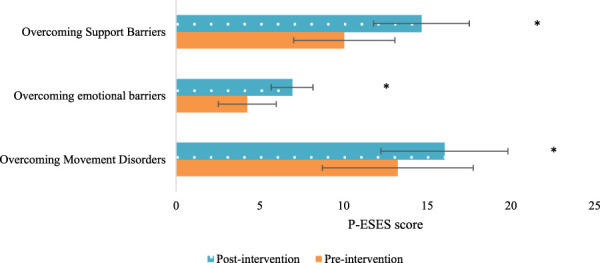
Comparison of the three dimensions of P-ESES scores after intervention in the two groups of patients (Note: * indicates marked difference).

The P-ESES score comprises three dimensions: overcoming exercise barriers, overcoming emotional barriers, and overcoming support barriers. After the intervention, the Exp group exhibited drastically superior scores in all three dimensions relative to the Con group (*P* < 0.05). This indicates that multidimensional quantitative exercise management has a positive impact on improving pregnant women’s ability to overcome exercise, emotional, and support barriers (Figure 2).

### 3.2 Analysis of blood glucose indicators

**FIGURE 3:**
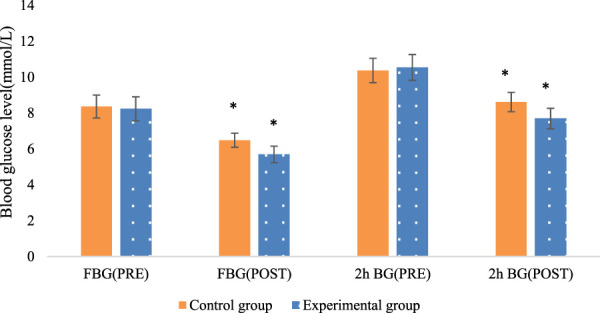
Comparison of fasting blood glucose and postprandial 2-h blood glucose levels before and after intervention in the two groups of patients (Note: * indicates marked difference).

The fasting blood glucose and postprandial 2-h blood glucose levels were compared between the two patient groups. There were neglectable differences between the groups before the intervention (*P* > 0.05). Nevertheless, after the intervention, there were marked differences within each group, with both fasting blood glucose and postprandial 2-h blood glucose levels substantially inferior to their respective pre-intervention levels. This indicates that timely exercise can effectively reduce the overall blood glucose levels in stable pregnant women. When comparing between the groups, the Exp group exhibited greatly lower blood glucose levels relative to the Con group (*P* < 0.05). This demonstrates the superior efficacy of multidimensional quantitative exercise management in controlling blood glucose levels (Figure 3).

**FIGURE 4:**
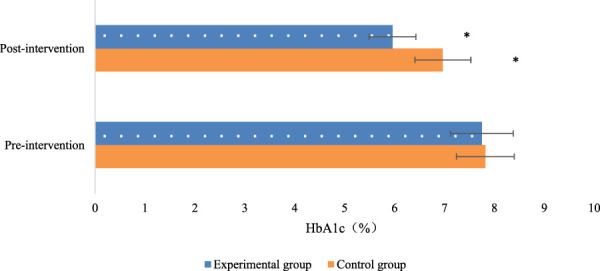
Comparison of glycated hemoglobin levels before and after intervention in the two groups of patients (Note: * indicates marked difference).

The levels of HbA1c were compared between the two patient groups. Within each group, there were notable reductions in HbA1c levels (*P* < 0.05), indicating that both groups experienced a decrease in HbA1c levels after the intervention. When comparing between the groups, Exp group, which underwent multidimensional quantitative exercise management, exhibited greatly lower HbA1c levels relative to Con group (*P* < 0.05). This suggests that multidimensional quantitative exercise management has a lowering effect on HbA1c levels in patients with GDM (Figure 4).

### 3.3 Analysis of delivery outcomes and neonatal complications

**FIGURE 5:**
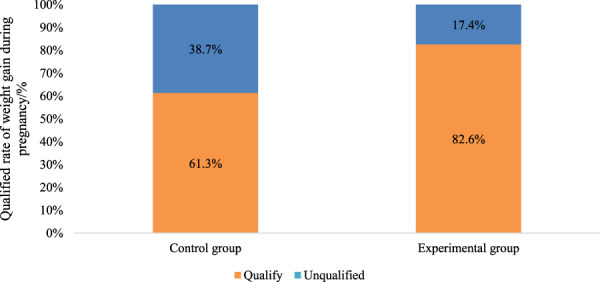
Comparison of the rate of achieving target weight in the two groups of patients.

The rate of achieving weight gain within the recommended range was significantly higher in the Exp group (82.6%) compared to the Con group (*P* < 0.05). This indicates that multidimensional quantitative exercise management has a notable advantage in controlling patient weight, effectively assisting patients in managing their weight, and ensuring a healthy pregnancy (Figure 5).

**FIGURE 6:**
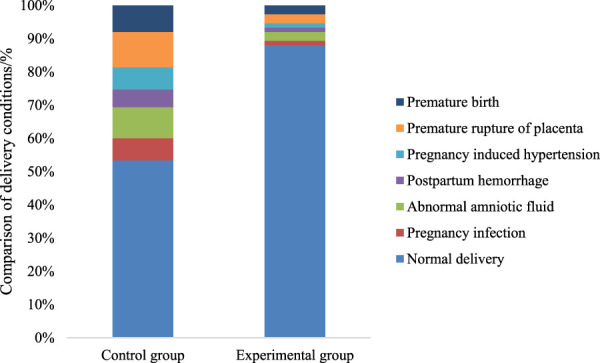
Comparison of delivery outcomes between the two groups of patients.

The comparison of delivery outcomes was conducted between the two patient groups. The Exp group had a drastically superior number of normal deliveries relative to the Con group. Both groups experienced cases of abnormal amniotic fluid, postpartum hemorrhage, and gestational hypertension. Nevertheless, the incidence rates of these complications were lower in the Exp group versus the Con group (*P* < 0.05). This demonstrates that multidimensional quantitative exercise management can effectively reduce the occurrence of delivery complications (Figure 6).

**FIGURE 7:**
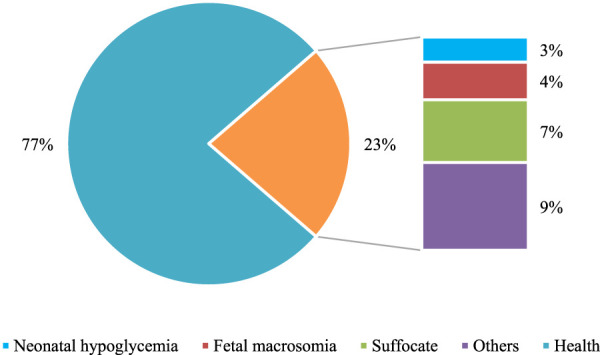
Proportional representation of the health status of newborns in Con group.

**FIGURE 8:**
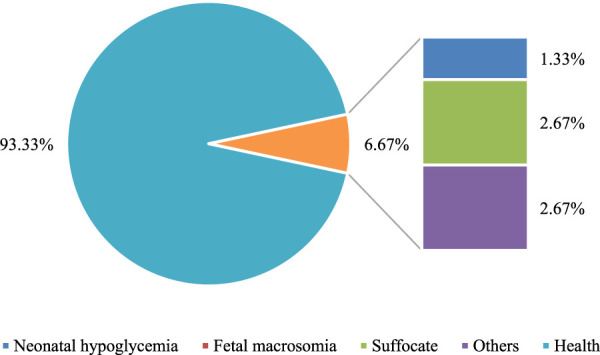
Proportional representation of the health status of newborns in Exp group.

The comparison of neonatal health status was conducted between the two groups. In the Con group, healthy newborns accounted for 77% of the total, while in the Exp group, healthy newborns accounted for 93.3% of the total. The data in the Exp group were remarkably superior to those in the Con group (*P* < 0.05). Both groups had varying degrees of symptoms such as asphyxia and hypoglycemia in newborns. Nevertheless, the incidence rate in the Exp group was substantially inferior to that in the Con group. Additionally, the Exp group had a 0% incidence rate of macrosomia, demonstrating a notable advantage of multidimensional quantitative exercise management in reducing neonatal complications (*P* < 0.05). Detailed data can be found in Figures 7, 8.

### 3.4 Analysis of patient VAS scores and Newborn Apgar scores

**FIGURE 9:**
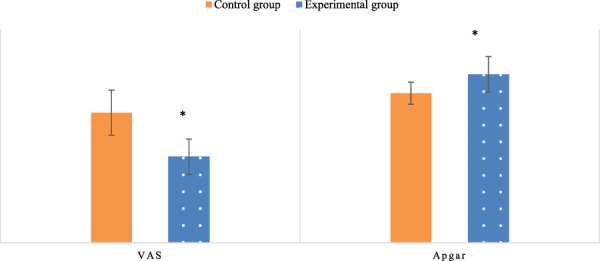
Comparison of VAS scores in the two groups of patients and Apgar scores of newborns.

The postpartum VAS scores and newborn Apgar scores were compared between the two groups. In the Exp group, the VAS score after multidimensional quantitative exercise management was 4.83, which was substantially inferior to the Con group (*P*< 0.05). This indicates that multidimensional quantitative exercise management had a positive effect on improving the overall physical wellbeing of patients. Newborn Apgar scores were significantly higher in the Exp group (9.47) compared to the Con group (*P* < 0.05), suggesting improved newborn health outcomes associated with multidimensional quantitative exercise management (Figure 9).

## 4 Discussion

Gestational Diabetes Mellitus (GDM) represents a significant health concern during pregnancy, characterized by elevated blood glucose levels due to insulin resistance exacerbated by hormonal changes (Anastasiou et al., 2020; Garmendia et al., 2020; Muche et al., 2020; Dipla et al., 2021). This condition not only poses risks to the mother’s health but also impacts fetal development, increasing the likelihood of complications such as macrosomia and respiratory distress syndrome (Feng et al., 2020b; Moon et al., 2022). Traditional management approaches for GDM typically involve dietary control, increased physical activity, and, in some cases, insulin therapy (Zhang et al., 2022).

However, recent studies have explored the efficacy of multidimensional quantitative exercise management as an alternative or complementary intervention to improve outcomes for pregnant women with GDM (Li et al., 2020; Karavasileiadou et al., 2022; Valadan et al., 2022). Our study aimed to investigate the effects of multidimensional exercise management on self-efficacy, blood glucose levels, and delivery outcomes in pregnant women diagnosed with GDM. The rationale behind this approach lies in its potential to address the underlying factors contributing to GDM, including insulin resistance and hormonal fluctuations. By implementing personalized exercise plans, real-time monitoring, and social support mechanisms, multidimensional exercise management seeks to empower pregnant women to better manage their condition and improve their overall health outcomes.

Our findings revealed significant improvements in self-efficacy among participants undergoing multidimensional exercise management compared to those receiving standard GDM care (Karavasileiadou et al., 2022). Self-efficacy, which refers to an individual’s belief in their ability to achieve desired outcomes, plays a crucial role in managing chronic conditions like GDM. By providing personalized feedback and monitoring tools, multidimensional exercise management interventions enhance participants’ confidence in their ability to control their blood glucose levels and adhere to recommended lifestyle modifications.

Furthermore, our study demonstrated a notable reduction in postprandial blood glucose levels among women in the multidimensional exercise management group compared to those in the standard care group (Li et al., 2020; Valadan et al., 2022). This finding suggests that structured exercise programs tailored to individual needs can effectively contribute to glycemic control in pregnant women with GDM. By incorporating aerobic exercise recommendations and personalized guidance, multidimensional exercise management interventions address the increased demand for insulin during pregnancy and help mitigate the adverse effects of insulin resistance on blood glucose levels.

The positive effects of multidimensional exercise management can be attributed to several key factors. Firstly, personalized monitoring and feedback mechanisms enable participants to track their exercise levels and blood glucose readings in real-time, facilitating greater awareness and self-regulation (Karavasileiadou et al., 2022). By empowering pregnant women with the tools and information needed to make informed decisions about their health, multidimensional exercise management interventions foster a sense of autonomy and self-efficacy. Secondly, the provision of tailored exercise plans based on individual physical condition and health goals encourages participants to engage in regular physical activity (Valadan et al., 2022). Aerobic exercise has been shown to improve insulin sensitivity and glucose uptake in skeletal muscles, making it an essential component of GDM management. By prescribing specific exercise regimens and providing ongoing support, multidimensional exercise management interventions help overcome barriers to physical activity participation and promote adherence to recommended guidelines.

Additionally, the establishment of social support networks facilitates peer-to-peer communication and information sharing among participants (Karavasileiadou et al., 2022). By creating a supportive environment where pregnant women can exchange experiences, seek advice, and provide encouragement, multidimensional exercise management interventions foster a sense of community and belonging. This social support network not only enhances participants’ motivation to engage in healthy behaviors but also provides emotional reassurance and validation.

Beyond its application in GDM management, multidimensional exercise management holds promise for addressing a range of other chronic conditions and promoting overall health and wellbeing (Muche et al., 2020; Belloni et al., 2021; Doré et al., 2022; Kouloutbani et al., 2023; Zhang et al., 2023). Studies have shown that structured exercise programs can improve mental health outcomes, enhance quality of life, and reduce the risk of developing chronic diseases such as type 2 diabetes and cardiovascular disease. By emphasizing the importance of regular physical activity and providing tailored support, multidimensional exercise management interventions offer a holistic approach to health promotion and disease prevention. However, it is essential to acknowledge the limitations of our study. The use of a single-center research design and a relatively small sample size may limit the generalizability of our findings. Future research should aim to replicate our findings in larger, multi-center studies involving diverse populations. Additionally, while our study focused primarily on the effects of exercise management on self-efficacy and blood glucose control, future studies could explore the broader impact of multidimensional interventions on other health outcomes, such as maternal-fetal health and delivery outcomes.

## 5 Conclusion

In conclusion, leveraging the multidimensional quantitative exercise management plan is an important direction in increasing outcomes in pregnant women with GDM. This intervention showed that patients had improvements in their self-efficacy as well as postprandial blood glucose concentration compared to the patients who received standard GDM care. The continual prompting and supervision that comes with multidimensional exercise management; the development of individual and specific exercise regimes; and the organized support networks strengthen women’s ability to combat their condition, additionally enhancing their general well-being. In addition, it is also pertinent to mention that the actual potential of this approach of not only useful in the management of GDM but also in other chronic diseases and disease prevention initiatives in general. Our work demonstrates the utility of multidimensional exercise management but subsequent studies need to build upon the work by limiting the study’s inherent weaknesses and investigating the general ability of our findings on the mater no-fetal health profile and delivery outcomes in even larger multi-centre trials. Altogether, the strategy of multidimensional exercise management is effective in regulating GDM as well as helping patients turn to optimal physical activity during pregnancy.

## Data Availability

The original contributions presented in the study are included in the article/Supplementary Material, further inquiries can be directed to the corresponding author.
